# Medical Journalism

**Published:** 1880-02

**Authors:** 


### Editorial.

### MEDICAL JOURNALISM.

In the days of the poet Juvenal there were men afflicted with a malady which he called *insanabile scribendi cacoethes*, and this scribbling cachexia—if it may be so translated—might appear to be prevalent in modern times among the members of our profession.

The first number of the *Index Medicus* contained a list of seven hundred and sixty-seven periodicals, relating to medicine or its collateral sciences. But all of these, whether modest or pretentious, may, in general, be divided into two classes—those for recording original observation, and those for retailing it to the professional at large. These two forms are often included in the same publication, but they are none the less entirely distinct. In Tyndall's lectures on "Light," he says:

"Three classes of workers are necessary; firstly, the investigator of natural truth, whose vocation it is to pursue that truth, and extend the field of discovery for the truth's own sake and without reference to practical ends; secondly, the teacher of natural truth, whose vocation it is to give public diffusion to the knowledge already won by the discoverer; thirdly, the applier of natural truth, whose vocation it is to make scientific knowledge available for the needs, comforts and luxuries of civilized life."

The same division of scientific labor exists to a certain extent in medicine. Men occupied in investigation alone, are comparatively few, and the elaborate details of method and reasonings often lie buried in ponderous volumes of "society transactions," "archives" and "annals." These are written principally for specialists, and in general are read by them alone. For example, there are twenty-seven periodicals devoted exclusively to anatomy and physiology, nineteen to obstretics, five to gynecology, eighteen to ophthalmology, forty-nine to "state medicine," sanitary and legal to a proportionate number to other branches.

The papers contributed to these form the cream of medical literature, and yet the busy physician seldom sees them. What he demands, is a convenient digest of the most practical points, and he finds it in our typical American Medical Journal. Thus it serves as the medium of communication between what Tyndall calls the "investigator" and the "applier" of natural truth.

A limited proportion of original communications are often acceptable, as are translations or clinical notes, but the selections, if good, are usually read first. As a whole, however, a journal is simply the medium, through which the medical thought of the vicinity finds expression.

Unless the professional standard of the locality is high—unless there be physicians acute in observing, careful in recording, and with sufficient zeal to express their opinions formally, a publication of this sort must inevitably languish and die.

It would, indeed, be a remarkable corps of editors who would, at stated periods, evolve from their own consciousness such mental pabulum, as would be invariably acceptable to a large class of readers. The few subjects, in which the individual writers might be interested, would necessarily be thrust upon the subscribers in repeated doses—*ad nauseam*—and simply result in establishing the egotism of the editor of that department.

The managers of this publication therefore cordially invite contributions of original articles, reports of cases, or other matter of interest to physicians; hoping thus, to add to its value professionally, to its interest locally, and trusting that the same elevated standard may be maintained, which, in former years has been reached by the BUFFALO MEDICAL AND SURGICAL JOURNAL.

